# *Campylobacter* gastroenteritis in children in north-eastern Israel comparison with other common pathogens

**DOI:** 10.1038/s41598-020-62744-y

**Published:** 2020-04-02

**Authors:** Waheeb Sakran, Zufit Hexner-Erlichman, Ronen Spiegel, Hamed Batheesh, Raphael Halevy, Ariel Koren

**Affiliations:** 1 Pediatric Department “B”, Emek Medical Center, Afula, Israel; 2 The Ruth and Baruch Rappaport School of Medicine, Technion, Haifa Israel; 3 Pediatric Hematology Unit, Emek Medical Center, Afula, Israel

**Keywords:** Pathogens, Bacterial infection

## Abstract

Gastroenteritis is common among children. *Campylobacter jejuni* is one of the main causative bacterial pathogens, together with *Shigella*, *Salmonella* and invasive *Escherichia coli*. Campylobacteriosis is a zoonotic, usually self-limited disease that does not always require antibiotic treatment. In cases of protracted diarrhoea in healthy children or immunocompromised patients, antibiotic treatment is recommended, and the drug of choice is still macrolides, with very low resistance rates in *Campylobacter* species. However, it is crucial to isolate the causative organism, because some cases, such as *Shigella* encephalitis, call for initiation of empiric antibiotic treatment. In this study, we compared the incidence, epidemiology, clinical findings and laboratory results of gastroenteritis with dysentery caused by these organisms in children in our area. *C. jejuni* was found to be the leading pathogen in children hospitalized with bacterial gastroenteritis, followed by *Shigella* and *Salmonella*. Macrolides were the drug of choice for *Campylobacter*, and ceftriaxone and ciprofloxacin were the best empiric treatments for *Shigella* and *Salmonella*, respectively.

## Introduction

Acute bacterial gastroenteritis is a common disease in infants and children. The clinical presentation includes fever, diarrhoea, bloody stool, vomiting, dehydration and abdominal pain.

*Campylobacter* species, *Shigella*, *Salmonella* and invasive *Escherichia coli* are the main bacterial organisms causing acute infection in the gastrointestinal tract^1^. *Campylobacter* organisms are thin curved Gram-negative non-spore-forming rods. The *Campylobacter* group includes 26 species: the most frequent types that cause disease in humans are *Campylobacter jejuni*, *Campylobacter coli* and *Campylobacter fetus*^2^, these are also the most common types in Israel^3^.

Campylobacteriosis is a zoonotic disease, with wild birds, poultry and domestic pets as the main reservoirs. Outbreaks are often linked to contaminated water, unpasteurized milk products, consumption of under-cooked chicken, environmental exposure and contact with farm animals^4^. It is difficult to differentiate acute gastroenteritis caused by *Campylobacter* from gastroenteritis caused by *Shigella* or *Salmonella* based only on clinical signs or routine blood analysis; stool culture provides the definitive diagnosis of the causative organism. In cases of severe dehydration, toxic appearance, signs of sepsis and gastroenteritis caused by *Shigella* or *Salmonella*, initiation of empiric antibiotic treatment, such as third-generation cephalosporin, is required; in cases suspected of *Shigella* encephalitis, empiric treatment is also recommended. Third-generation cephalosporin has no effect against *Campylobacter*^4^.

*Campylobacter* is considered the causative organism in most cases of bacterial gastroenteritis in young children. It is usually a self-limited disease and does not always require antibiotic treatment. In cases of protracted diarrhoea in healthy children, or infection in immunocompromised patients, antibiotic treatment is recommended, and the drug of choice is still macrolide antibiotics, with very low resistance rates in *Campylobacter* species^5^.

The goals of the present study were to examine whether *C. jejuni* is the leading pathogen of acute gastroenteritis in children admitted with diarrhoea and bloody stool to paediatric departments, and to compare the demographic data, clinical findings and routine blood analysis results to the same data in patients presenting with other pathogens that cause a similar disease.

## Methods

We searched for children who were hospitalized between January 2003 and December 2012 at our medical centre in the north of Israel, which serves a population of Jewish, and Muslim and Christian Arab origins. The patients were admitted to the paediatric department with main complaints of acute gastroenteritis, bloody stools or signs of dysentery. We included only patients with positive stool cultures. Routine policy is to obtain blood cultures from patients who suffer from fever during presentation or hospitalization. We analysed demographic data, and clinical signs, such as fever, vomiting and dehydration. We compared these data from cases with positive stool culture for *Campylobacter* spp. to those with other pathogens that cause bloody diarrhoea in the same cohort. At our centre, we do not perform antibiograms for *Campylobacter* that causes acute gastroenteritis. For the other organisms, we test for sensitivity patterns to various antibiotics. We explored other complications, such as bacteraemia, and we also looked for the treatment received by the patient. The purpose of our study was to compare the incidence of *Campylobacter* as a cause of bloody diarrhoea with that of other groups of bacteria that cause a similar disease.

The stool culture was performed as follows. A bean-sized stool specimen was suspended in 2 ml of a 0.9% NaCl solution and used to inoculate one half of a *Salmonella Shigella* (SS) agar plate and a *Campylobacter* selective agar plate. The saline stool suspension was then overlaid with Mueller Kaufmann brilliant green bile enrichment broth. *Campylobacter* plates were incubated in a microaerophilic atmosphere in a jar at 42 °C, while the SS plates were incubated overnight at 37 °C. On the following day, the second half of each SS plate was inoculated with a loop full of the appropriate specimen from the surface of the Mueller Kaufmann brilliant green broth. Suspected *Shigella*/*Salmonella* colonies were picked and identified using Kligler enteric test tubes, mass spectrometry (Bruker), and antiserum agglutination. Each culture was incubated for a total of 48 h. *Campylobacter* plates were evaluated after 24, 48 and 72 h of incubation. Identification was performed using Gram stain according to the Bruker Mass Spectrometry Clinical Microbiology Procedure Handbook (Lynne S. Garcia *et al*., 2007 update).

All experiments were performed in accordance with the relevant guidelines and regulations. The study received approval from the Helsinki Committee of Emek Medical Center, approval number: EMC-0143-12, issued on 6 Jan 2013. The committee waived the need for informed consent as part of the study approval, since this was a retrospective data analysis.

To analyze the differences between patient characteristics in the different bacterial groups, a series of *χ*^2^ tests or Fisher's exact tests (performed when the assumptions of the parametric *χ*^2^ test were not met) were conducted. To test whether the groups differed in continuous characteristics data, a linear model ANOVA or nonparametric Kruskal–Wallis rank-sum test (where the sample means did not satisfy the normality assumption) was conducted. The Bonferroni correction was used for multiple comparisons. We computed the two-tailed *P* values, where *P* < 0.05 was considered a statistically significant result. Statistical analyses were performed using R statistical software version 3.6.1.

## Results

Positive stool cultures were found in 622 patients, representing 18% of all patients hospitalized under the presumptive diagnoses listed in Methods, during the designated time period. Demographic characteristics of the patients according to the three common enteropathogens are presented in Table 1. Overall, 356 (57%) patients were male, 266 (43%) female, and 367 (59%) were of Arab origin. The ethnic distribution in the paediatric population served by our medical centre is 64% Arab (Muslims and Christians) and 35% Jewish. We found significant differences in age between the *Campylobacter* and *Shigella* groups and between the *Shigella* and *Salmonella* groups (*P* < 0.001 for both), and no significant difference between the *Campylobacter* and *Salmonella* groups (Table 1).

**Table 1:** Demographic characteristics of children with *Campylobacter*, *Shigella* and *Salmonella* in north-eastern Israel, 2003–2012.

Parameters		*Campylobacter* (N = 332) 1	*Shigella* (N = 158) 2	*Salmonella* (N = 132) 3	*P*	*P* for multiple comparisons performed	
Gender N (%)	Males	196 (59)	83 (53)	77 (58)	0.38		
Age (mo)	Mean (±s.d.)	36.1 (57.0)	72.2 (47.4)	26.7 (40.5)	<0.001	1 vs. 2	<0.001
	Median	12.5	63	12		1 vs. 3	0.999
	Q1, Q3	5, 27	37, 95	5, 22.25		2 vs. 3	<0.001
Ethnicity	Arab origin	323 (70.2%)	55 (34.8%)	79 (59.8%)	<0.001		
	Jewish origin	99 (29.8%)	103 (65.2%)	53 (40.2%)			

Three groups of bacteria were found in the present study: *Campylobacter* was isolated from 53.3% of the samples (Table 1). Of these, 288 (86.74%) were *C. jejuni* isolates, 15 (4.51%) *C. coli* isolates, and 29 (8.37%) *Campylobacter* species isolates (our laboratory does not specify the isolates beyond those mentioned). The second group included patients with *Shigella*; of the isolates found, 145 (91.7%) were *Shigella sonnei*, followed by 8 (5.7%) *S. flexneri* and 4 (2.5%) *S. boiydee* isolates. The *Salmonella* group included 21.2% of the patients; all of the isolates were *Salmonella enterica*.

Fever at admission, length of hospital stay and duration of diarrhoea are shown in Table 2, and significant differences were found between groups (Table 2). A comparison of mean temperatures between groups showed a significant difference in fever extent between *Campylobacter* and *Shigella* groups (*P* = 0.003). The average length of hospitalization also showed significant differences between groups (Table 2).

**Table 2:** Fever, and length of hospitalization and diarrhoea duration.

	Parameters	*Campylobacter* (N = 332) 1	*Shigella* (N = 158) 2	*Salmonella* (N = 132) 3	*P*	*P* for multiple comparisons performed	
Fever at admission (oC)	Mean (±s.d.)	38 (1.1)	38.4 (1.2)	38.2 (1.3)	<0.005	1 vs. 2	0.003
	Median	38	38.5	38.1		1 vs. 3	0.66
	Q1, Q3	37.2, 38.9	37.7, 39.3	37.3, 39.2		2 vs. 3	0.33
Length of hospital stay (days)	Mean (±s.d.)	4.343 (3.7)	3.6 (1.3)	5.4 (3.8)	<0.001	1 vs. 2	0.005
	Median	4	3	4		1 vs. 3	<0.001
	Q1, Q3	3, 5	3, 4	4, 6		2 vs. 3	<0.001
Duration of diarrhoea (days)	Mean (±s.d.)	5.3 (5.1)	3.5 (1.4)	6.2 (5)	<0.001	1 vs. 2	<0.001
	Median	4	3	5		1 vs. 3	0.003
	Q1, Q3	3, 6	3, 4	3, 7		2 vs. 3	<0.001

Mean duration of diarrhoea was significantly different between groups (Table 2). We searched for the presence of diarrhoea and bloody diarrhoea in the three groups, but there was no significant difference between the groups. In the *Campylobacter*, *Shigella* and *Salmonella* groups, 67%, 88% and 83% of the patients, respectively, received intravenous fluids.

A total of 1320 blood cultures were obtained during the research period from patients included in the study. Four patients were diagnosed with uncomplicated bacteraemia: *S. enterica* and two isolations of *C. jejuni*. In one patient, *Staphylococcus coagulase* negative was isolated and, in another patient, a *Bacillus* species was isolated; these two pathogens were considered to be contaminants.

At our institution, we do not perform antibiograms for *Campylobacter* isolated from stool. The *Shigella* isolates were sensitive to ciprofloxacin, ceftriaxone and nalidixic acid (Table 3). The *Salmonella* organisms were sensitive to ceftriaxone and ciprofloxacin (Table 3). The *Salmonella* isolates were not significantly more sensitive to those antibiotics (*P* < 0.001 for both drugs) than the *Shigella* isolates (Table 3)

**Table 3:** Sensitivity patterns of *Shigella* and *Salmonella* groups to various antibiotics.

Antibiotics	*Shigella* group (158 isolates)			*Salmonella* group (132 isolates)			*P*-value (x^2^)
	Number of isolates	Number of sensitive isolates	Sensitivity (%)	Number of isolates	Number of sensitive isolates	Sensitivity (%)	
Amoxicillin + clavula*	143	91	64	116	101	87	NS
Ampicillin	151	40	26	120	95	79	<0.001
Ciprofloxacin	155	154	99	122	118	97	NS
Ceftriaxone	155	148	95	121	120	99	NS
Nalidixic acid	155	145	94	119	78	66	NS
Sulpha + trimethoprim**	154	15	10	122	97	80	<0.001
Tetracycline	152	114	75	114	60	53	NS

*Amoxicillin + clavulanic acid.

**Sulphamethoxazole and trimethoprim.

NS, not significant.

A total of 267 patients (43%) received antibiotic therapy before and/or after receiving the stool culture results; 87 patients were treated prior to the culture results, and 31 following the culture results. Some data were not available in the medical charts of some patients in the three group, as demonstrated in Table 4. In the *Campylobacter*, *Shigella* and *Salmonella* groups, 40%, 49% and 44% of the patients received antibiotic treatment. In the *Campylobacter* group, 60% of the patients received macrolides either before or after the culture results; 71% received ceftriaxone, which has no effect against *Campylobacter*; 19% were treated with a dual empiric antibiotic regimen that included azithromycin and ceftriaxone. Ceftriaxone was mostly used as the empiric treatment in severe cases, and only one patient in the *Campylobacter* group received ceftriaxone after the culture results arrived. The standard course of ceftriaxone treatment was a single dose per day for 3 days. In the *Shigella* and *Salmonella* groups, 82% and 83% of the patients, respectively, received ceftriaxone. Most patients received antibiotics prior to the culture results. As shown in Table 4, there was no significant difference between the number of treatment days before and after culture results among the three groups.

**Table 4:** Days of antibiotic treatment before and after culture results in the three groups.

	Parameters	*Campylobacter* (N = 70)	*Shigella* (N = 28)	*Salmonella* (N = 110)	*P*
Treatment before culture results (days)	N	55	22	10	0.588
	Mean (±s.d.)	3.1 (1.2)	3 (0.5)	2.9 (0.3)	
	Median	3	3	3	
	Q1, Q3	3, 3	3,3	3, 3	
Treatment after culture results (days)	N	22	7	2	0.404
	Mean (±s.d.)	4.4 (3.3)	3 (0)	2 (0)	
	Median	3	3	3	
	Q1, Q3	3, 3	3, 3	3, 3	

This table includes information concerning patients in the three pathogen groups, which was obtained from their medical charts.

A significant difference was found in the length of the hospital stay (*P* < 0.001) and diarrhoea duration (*P* = 0.003) in patients who were treated with antibiotics compared to those who were not treated. These parameters were higher in the patients that were treated (Table 5).

**Table 5:** Length of hospital stay and duration of diarrhoea in treated vs. untreated patients.

	Parameters	No antibiotic treatment	Antibiotic treatment	*P*
Length of hospital stay (days)	Mean (±s.d.)	3.9 (1.5)	5 (4.8)	<0.001
	Median	4	4	
	Q1, Q3	3, 5	3, 5	
Duration of diarrhoea (days)	Mean (±s.d.)	4.5 (3.8)	5.7 (5.3)	0.003
	Median	4	4	
	Q1, Q3	3, 5	3, 6	

## Discussion

*Campylobacter* species are an important cause of bacterial gastroenteritis, especially among hospitalized children, followed by *Shigella* and *Salmonella*. These pathogens should be considered in children who suffer from sudden onset of protracted diarrhoea or have symptoms that appear more toxic than a state of dehydration. Systemic, extra-intestinal dissemination of these pathogens is uncommon, with the exception of *Salmonella* infection in immunocompromised hosts or during the first year of life. Complications such as bacteraemia are not common in *Campylobacter* or *Shigella* infections, but have been described in infants with *Salmonella*, especially *Salmonella typhi*. These pathogens may cause neurological complications such as: seizures in *Shigella* and meningitis in *Salmonella* and *Campylobacter*^1,2,4,5^. *Salmonella* may also lead to pneumonia and myocarditis. *Campylobacter* may cause urinary tract infections and cholecystitis^6^.

As shown here and by others^1,4,5,7^, *Shigella* is more common in older children and adults, whereas *Campylobacter* and *Salmonella* are more frequently found in younger children and infants. *C. jejuni* is the most frequently isolated *Campylobacter* species in cases of gastroenteritis, and it is the most common organism causing foodborne illnesses in the United States.

Generally, *Campylobacter* infections are mild, mostly self-limited and require only supportive care. Nevertheless, some cases may lead to severe dehydration. Severe cases of protracted diarrhoea and infection in immunocompromised children and young infants may benefit from antibiotic treatment^4–7^. Our study did not show a shortened duration of hospitalization or diarrhoea among patients that received antibiotics, possibly due to a more severe course of disease among patients whose condition necessitated antibiotic therapy.

The drugs of choice were found to be ceftriaxone and azithromycin, either alone or as a dual therapy. Ceftriaxone was used mostly as an empiric therapy in severe cases, whereas azithromycin was used as empiric and specific therapy following culture results. We do not perform antibiograms for *Campylobacter* stool isolates. As mentioned in previous reports, less than 5% of *Campylobacter* spp. are resistant to macrolides^5,7,8^. Nonetheless, in cases of serious infection, where there is no response to macrolide treatment, drug sensitivity studies should be performed. Recent studies conducted in Asia and the United States reported increasing rates of *Campylobacter* resistance to macrolides as well as fluoroquinolones^7^. The sensitivity of *Shigella* and *Salmonella* isolates to ciprofloxacin and ceftriaxone, was not significant in our study. It is important to mention that, ciprofloxacin is not the drug of choice for children due to arthropathy.

Complications caused by *Campylobacter* gastroenteritis are rare, although early- and late-onset complications have been described. Early-onset complications include: septic arthritis, bursitis, osteitis, soft tissue infections, erythema nodosum, glomerulonephritis, haemolytic anaemia, IgA nephropathy, post-infectious irritable bowel syndrome, and intestinal perforation. *Campylobacter* is also the most commonly identified cause of Guillain-Barré syndrome and can cause reactive arthritis as a late-onset complication^6^.

*Campylobacter* can also cause invasive diseases, including bacteraemia. We found two cases of bacteraemia due to *Campylobacter*. Very few previous studies have described *Campylobacter* bacteraemia in Israel or elsewhere^9–11^. Bacteraemia is a rare finding of *Campylobacter* gastroenteritis in immunocompetent children, but it has been found in several cases of immunocompromised children. The outcome is usually favourable, with the administration of broad-spectrum antibiotic therapy, such as carbapenems, gentamicin or amikacin.

Studies conducted in Peru^12^ Pakistan^13^ and Thailand^14^ have demonstrated results similar to those found here. In those areas, *Campylobacter* was the major cause of dysentery among children under the age of 5 years, followed by *Shigella* and other enteropathogens. In our study, campylobacteriosis was found to be more common among infants and young children, and *Shigellosis* was seen in older children. *Campylobacter* isolates were sensitive to macrolides, whereas *Shigella* isolates were sensitive to third-generation cephalosporin and quinolones.

In conclusion, *C. jejuni* is one of the most common causes of bacterial gastroenteritis in young hospitalized children, and is usually self-limited. Severe cases should be treated with antibiotics, usually macrolides, although increasing rates of resistance, especially to macrolides and fluoroquinolones, should be considered. Even though complications are rare, it is important to isolate the causative organism from the stool, to initiate the appropriate therapy.

### Ethical approval

This study received approval from the Helsinki Committee of the Emek Medical Center, approval number: EMC-0143-12, issued on 6 Jan 2013.

## Data Availability

All data generated or analysed during this study are included in this published article.
